# High-Density Lipoprotein Cholesterol, Blood Urea Nitrogen, and Serum Creatinine Can Predict Severe Acute Pancreatitis

**DOI:** 10.1155/2017/1648385

**Published:** 2017-08-22

**Authors:** Wandong Hong, Suhan Lin, Maddalena Zippi, Wujun Geng, Simon Stock, Vincent Zimmer, Chunfang Xu, Mengtao Zhou

**Affiliations:** ^1^Department of Gastroenterology, The First Affiliated Hospital of Soochow University, Suzhou, Jiangsu, China; ^2^Department of Gastroenterology and Hepatology, The First Affiliated Hospital of Wenzhou Medical University, Wenzhou, Zhejiang, China; ^3^Unit of Gastroenterology and Digestive Endoscopy, Sandro Pertini Hospital, Rome, Italy; ^4^Department of Anesthesiology, The First Affiliated Hospital of Wenzhou Medical University, Wenzhou, Zhejiang, China; ^5^Department of Surgery, World Mate Emergency Hospital, Battambang, Cambodia; ^6^Department of Medicine II, Saarland University Medical Center, Kirrberger Str., 66421 Homburg, Germany; ^7^Department of Medicine, Marienhausklinik St. Josef Kohlhof, Neunkirchen, Germany; ^8^Department of Surgery, The First Affiliated Hospital of Wenzhou Medical University, Wenzhou, Zhejiang, China

## Abstract

**Background and Aims:**

Early prediction of disease severity of acute pancreatitis (AP) would be helpful for triaging patients to the appropriate level of care and intervention. The aim of the study was to develop a model able to predict Severe Acute Pancreatitis (SAP).

**Methods:**

A total of 647 patients with AP were enrolled. The demographic data, hematocrit, High-Density Lipoprotein Cholesterol (HDL-C) determinant at time of admission, Blood Urea Nitrogen (BUN), and serum creatinine (Scr) determinant at time of admission and 24 hrs after hospitalization were collected and analyzed statistically.

**Results:**

Multivariate logistic regression indicated that HDL-C at admission and BUN and Scr at 24 hours (hrs) were independently associated with SAP. A logistic regression function (LR model) was developed to predict SAP as follows: −2.25–0.06 HDL-C (mg/dl) at admission + 0.06 BUN (mg/dl) at 24 hours + 0.66 Scr (mg/dl) at 24 hours. The optimism-corrected c-index for LR model was 0.832 after bootstrap validation. The area under the receiver operating characteristic curve for LR model for the prediction of SAP was 0.84.

**Conclusions:**

The LR model consists of HDL-C at admission and BUN and Scr at 24 hours, representing an additional tool to stratify patients at risk of SAP.

## 1. Introduction

Most patients with acute pancreatitis (AP) present a mild course while 10%–20% of them develop severe disease with significant mortality [1]. Early prediction of disease severity of AP would be helpful for triaging patients to the appropriate level of care and intervention [2]. It was reported that clinical scoring systems such as Harmless Acute Pancreatitis Score (HAPS) and Bedside Index for Severity in Acute pancreatitis (BISAP) show moderate diagnostic accuracy in the prediction of SAP [3]. Simple, routine, and widespread individual laboratory parameters, such as Blood Urea Nitrogen (BUN) and serum creatinine (Scr), have been proposed as markers of disease severity [4]. However, to the best of our knowledge, information about serum creatinine at 24 hours after admission as a predictor of SAP in the literature is limited [5].

It was noticed that severe acute pancreatitis can alter serum lipid levels [6]. Recently, a small sample size (66 patients) study by Peng et al. [7] reported that low levels of High-Density Lipoprotein Cholesterol (HDL-C) are associated with high risk of persistent organ failure in AP. It should be stressed that only the patients hospitalized in Intensive Care Unit (ICU) were enrolled in the above study, thus limiting the possibility of applying their results to general ward and to community hospital.

On the other hand, these simple parameter variables demonstrate low sensitivity despite high negative predictive values for prediction of SAP [3]. It is hypothesized that combining several clinical parameters may improve predictive sensitivity [2].

Therefore, the primary aim of the study was to develop a multivariable model for prediction of SAP. The secondary one was to assess the ability of serum creatinine at 24 hours after admission and HDL-C as predictors of SAP in a large sample study.

## 2. Materials and Methods

### 2.1. Patient Population, Data Collection, and Ethics

A total of 647 patients suffering from acute pancreatitis that attended First Affiliated Hospital of Wenzhou Medical University between January 2013 and December 2015 were enrolled in the study. Exclusion criteria included [2, 5] onset time > 3 days, recurrent pancreatitis, organ failure before data collection, malignancy, previous pancreatic surgery, gestation, intoxication, endoscopic retrograde cholangiopancreatography (ERCP) or trauma-induced pancreatitis, chronic pancreatitis, AP in a moribund patient as a component of the terminal illness, and complete data being unavailable.

The following information was collected for each patient: age, gender, Body Mass Index (BMI), etiology, hematocrit, High-Density Lipoprotein Cholesterol (HDL-C) at admission. Blood Urea Nitrogen (BUN) and serum creatinine (Scr) were registered at the time of admission and 24 hrs after hospitalization.

This study protocol was approved by the Ethic Committee of the First Affiliated Hospital of Wenzhou Medical University and it was performed according to the principles expressed in the Declaration of Helsinki. Informed consent was obtained by the subjects.

### 2.2. Definitions and Outcomes

The diagnosis of AP requires two of the following three features in the revised Atlanta criteria [5, 8]: (1) abdominal pain; (2) level of serum amylase or lipase at least three times greater than the upper limit of normal; and (3) characteristic findings of AP on abdominal image. According to these criteria, mild AP is defined as no organ failure or systemic or local complications, while moderate/severe AP as one or more transient organ failure or systemic or local complications [1]. SAP consists of persistent organ failure for more than 48 hrs [1, 8]. Organ failure was detected through the Marshall score ≥ 2 for at least one of the three organs involved (cardiovascular failure, respiratory failure, and renal failure) [9]. Biliary AP was defined when gallstones or biliary sludge were observed on abdominal image. Alcohol was considered to be an etiological factor when patients had a history of alcohol consumption within 48 hours before symptom onset [10]. In the absence of gallstones and history of alcohol use, a serum triglyceride was considered the etiology if >1000 mg/dL [11]. The etiology was considered idiopathic if causative factors could not be identified after detailed clinical, laboratory, and imaging investigations.

The primary endpoint was to develop a logistic regression equation to predict severe acute pancreatitis. The secondary one was to assess the ability of serum creatinine at 24 hours after admission and validate HDL-C predictors of SAP at initial admission entering the hospital.

### 2.3. Statistical Analysis

A Shapiro-Wilk test was used to evaluate whether the continuous data showed a normal distribution. According to its results, continuous values were expressed by mean ± SD or median and Interquartile Range (IQR) and compared using one-way analysis of variance or the Kruskal-Wallis nonparametric test. Categorical values were described by count and proportions and compared by the *χ*^2^ test or Fisher's exact test.

Linear trend of categorical and continuous variables was tested by a Royston extension of the Cochran-Armitage test [12] and a nonparametric Wilcoxon rank sum test [13], respectively.

All the variables found to be different between patients with and without severe acute pancreatitis on univariate analysis were included as eligible factors in a forward-conditional stepwise logistic regression analysis. For this analysis, the conditional probabilities for stepwise entry and removal of a factor were 0.05 and 0.10, respectively [14]. Based on the results of multiple logistic regression analysis, a logistic regression equation model was developed to predict severe acute pancreatitis. Model calibration, reflecting the link between predicted and observed risk, was evaluated by plotting the predicted versus observed deciles of predicted risk and checked by Hosmer-Lemeshow goodness-of-fit test [15, 16]. Odds ratios (OR) were calculated, with 95% CI [17]. Multicollinearity was considered to be significant if the largest variance inflation factor exceeded 10 [2].

The area under the receiver operating characteristic (ROC) curve, that is, AUC, was used to evaluate the performance of predictions. A variable with an AUC above 0.7 was considered useful, while an AUC between 0.8 and 0.9 indicated excellent diagnostic accuracy [14]. The c-index, which is equivalent to the area under receiver operating characteristic curve, was also used to assess the predictive accuracy of the model [18]. Bootstrap resample technique with 1000 bootstrap replications, as a method of internal validation, was used to estimate an unbiased optimism-corrected estimate of c-index [19]. Optimism is estimated as the average of the difference in performance of the model in the bootstrap sample and original dataset [16].

As described by Maksimow et al. [20], the best cut-off point was detected where the number of false positives is the lowest as possible (specificity > 90%) by selecting a threshold value at a point where the longest increase in the sensitivity of the slope declines, since ICU beds are limited. The sensitivity, specificity, negative predictive value (NPV), and positive predictive value (PPV) were calculated for corresponding cut-off values.

Differences were considered to be statistically significant if the two-tailed *p* value was less than 0.05.

## 3. Results

### 3.1. Patient Characteristics

The median age of the 647 patients included in the study was 47 (IQR 37–63), of which 406 (62.8%) were male. Biliary disease was the most common cause of the AP (272/647, 42.0%). The median BISAP score at the time of hospital admission was 1. There were 491 (75.9%), 98 (15.2%), and 58 (8.9%) with mild, moderate/severe, and severe acute pancreatitis, respectively. Among the 58 patients who developed SAP, multiple organ failure was noted in 27 (46.6%) of them. Respiratory failure (84.5%) was the most frequent manifestation. The median length of the hospital stay was 10 days (IQR 7–14 days), with 15.5 days (IQR 10–28 days) for SAP patients. Ten patients (1.55%) died during hospitalization.

### 3.2. Univariable and Multivariable Analysis

As shown in Table 1, univariate analysis suggested that BMI, etiology, hematocrit, HDL-C at admission, and BUN and Scr at the time of admission and 24 hours after hospitalization were significantly associated with severe acute pancreatitis. A multivariate analysis was performed by a logistic regression for all these variables. HDL-C at admission (OR 0.95; 95% CI 0.92–0.97; *p* < 0.001), BUN at 24 hours (OR 1.07; 95% CI 1.03–1.11; *p* = 0.001); and Scr at 24 hrs (OR 1.94; 95% CI 1.03–3.66; *p* = 0.039) were independently associated with severe acute pancreatitis.

### 3.3. Model Development, Calibration, and Bootstrap Validation

**Figure 1:**
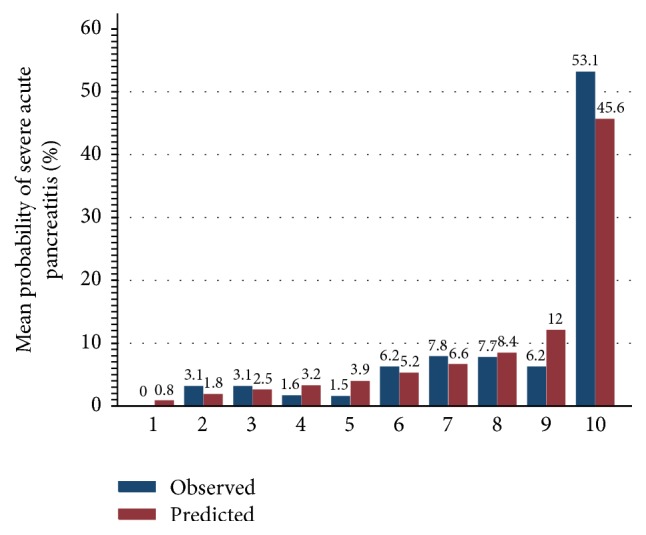
LR model calibration plot. Patients were ranked by their predicted probability and divided into 10 equal groups. The red bars represent the mean predicted probabilities for each of the 10 groups and blue bars represent the observed probabilities with severe acute pancreatitis in each of these same groups. LR model = logistic regression model.

A logistic regression function* (LR model)* was developed aiming to predict severe acute pancreatitis as follows: −2.25–0.06 HDL-C (mg/dl) at admission + 0.06 BUN (mg/dl) at 24 hours + 0.66 Scr (mg/dl) at 24 hours. As showed in Figure 1, calibration plots indicate adequate predicted probabilities against observed proportions of SAP. The Hosmer-Lemeshow goodness-of-fit test was also significant (*p* = 0.567), suggesting that our prediction model fits the actual data well. Bootstrap validation indicated that the estimated optimism is 0.018 and optimism-corrected c-index of 0.832.

### 3.4. Diagnostic Values of Various Predictors

**Figure 2:**
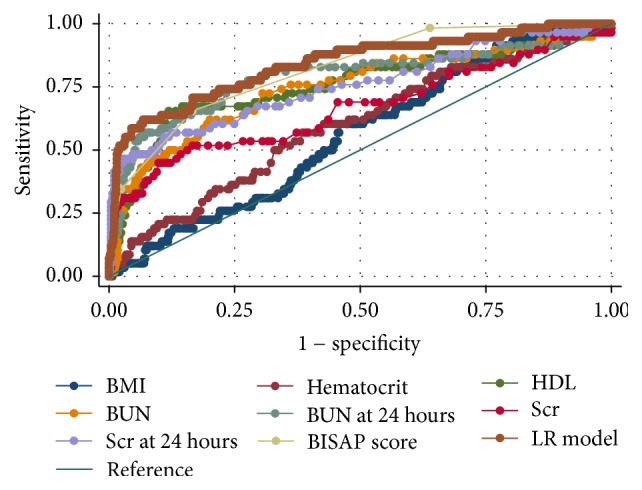
ROC curves for various predictors for severe acute pancreatitis. The AUCs for BMI at admission, hematocrit at admission, HDL-C at admission, BUN at admission, BUN after 24 hrs of admission, Scr at admission, Scr after 24 hrs of admission, BISAP score, and LR model for the prediction of SAP were 0.56 ± 0.04, 0.60 ± 0.04, 0.76 ± 0.04, 0.75 ± 0.04, 0.79 ± 0.04, 0.67 ± 0.05, 0.76 ± 0.04, 0.82 ± 0.03, and 0.84 ± 0.03, respectively. The ideal AUC was 1.00. The reference line represents AUC of 0.50, based on chance alone. ROC curve = receiver operating characteristic curve; AUC = area under the receiver operating characteristic curve; BMI = Body Mass Index; HDL-C = High-Density Lipoprotein Cholesterol; BUN = Blood Urea Nitrogen; Scr = serum creatinine; BISAP = Bedside Index for Severity in Acute pancreatitis; LR model = logistic regression model.

As shown in Figure 2, the AUCs for BMI at admission, hematocrit at admission, HDL-C at admission, BUN at admission, BUN after 24 hrs of admission, Scr at admission, Scr after 24 hrs of admission, BISAP score, and LR model for the prediction of SAP were 0.56 ± 0.04, 0.60 ± 0.04, 0.76 ± 0.04, 0.75 ± 0.04, 0.79 ± 0.04, 0.67 ± 0.05, 0.76 ± 0.04, 0.82 ± 0.03, and 0.84 ± 0.03, respectively. HDL-C, BUN at admission, BUN at 24 hours, and Scr after 24 hrs of admission were useful predictors of SAP, with AUC of more than 0.7. In addition, BISAP score and LR model showed to be excellent predictors of SAP, with an AUC of more than 0.8.

Based on the ROC curve analysis, the optimum cut-off values of HDL-C, BUN at admission, BUN at 24 hours, Scr after 24 hrs, BISAP score, and LR model were 22.4 (mg/dl), 22.7 (mg/dl), 21.8 (mg/dl), 1.02 (mg/dl), 2, and −1.86, respectively (Table 2).

**Figure 3:**
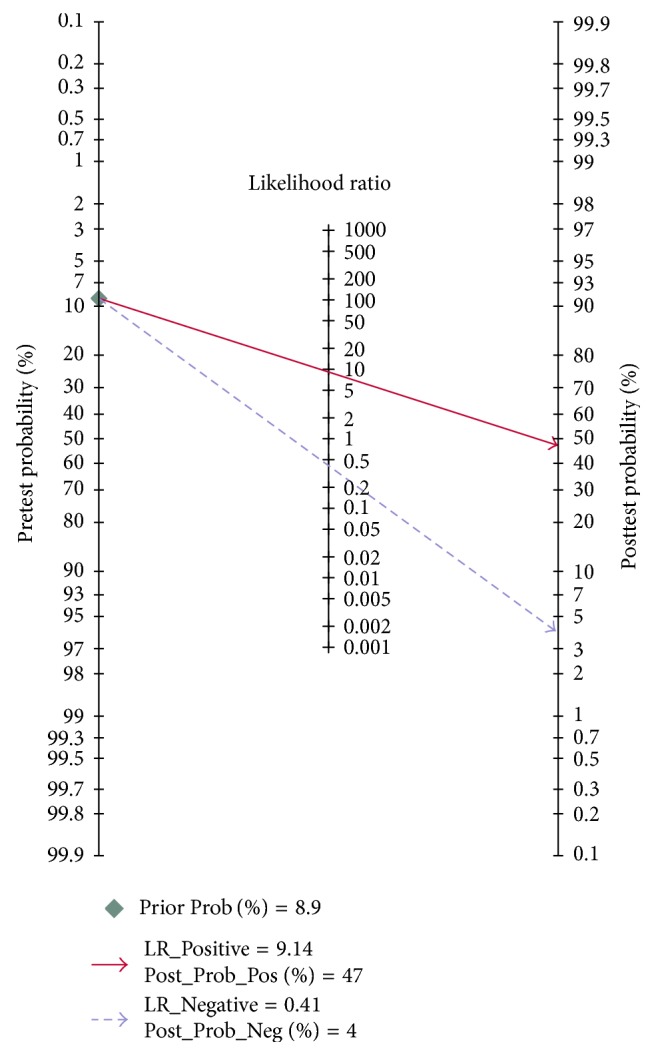
Fagan plot for LR model for prediction of severe acute pancreatitis. LR model = logistic regression model.

Using a cut-off value of −1.86 and the prevalence of organ failure in acute pancreatitis (8.9% in this study) as the pretest probability, the Fagan plot (Figure 3) shows that LR model can be clinically informative as it increases the probability of being classified into severe acute pancreatitis up to 47%, when positive, and lowers the probability to 4% when negative.

## 4. Discussion

The results of the present study demonstrated the following: (i) LR model and BISAP score were excellent predictors of SAP, with an AUC of more than 0.8 (Figure 2). The developed prediction model showed good predictive performance in terms of discrimination (optimism-corrected c-index: 0.832) and calibration (Hosmer-Lemeshow test: *p* = 0.567) (Figure 1); (ii) HDL-C, BUN at admission, BUN at 24 hours, and Scr after 24 hrs of admission were useful predictors of SAP, with an AUC of more than 0.7.

A rise in the BUN level reflects the disease status of initial intravascular volume depletion and prerenal azotemia in AP [2]. A body of evidences suggested that it is an important predictor for the assessment of SAP. An international validation study noted that a BUN level of 20 mg/dl or a higher one was associated with increased incidence of mortality (OR 4.6, 95% CI, 2.5–8.3) [21]. Koutroumpakis et al. [4] suggested that admission hematocrit ≥ 44% and rise in BUN at 24 hrs were the most accurate in predicting persistent organ failure (AUC: 0.67 and 0.71, resp.) and pancreatic necrosis (AUC: 0.66 and 0.67, resp.), outperforming the other laboratory parameters. Our study noted that BUN after 24 hours of hospitalization had higher AUC than that at initial admission (0.79 versus 0.75) (Figure 2). With a cut-off of 21.8 mg/dl, BUN at 24 hours of hospitalization achieved a sensitivity of 56.9%, specificity of 90.2%, PPV of 36.3%, and NPV of 95.5% (Table 2). The low PPV of BUN suggested that azotemia would recover to normal in a few patients if they could receive successful volume resuscitation. These results are consistent with previous observation according to which patients who experienced a decrease in the BUN level in hospitalizing had substantially reduced mortality [22].

Like BUN, Scr is also a marker of renal function. A rise in Scr reflects the disease states of initial hypovolemia and renal dysfunction in SAP and it represents an important factor for the assessment of severity [23]. Wilkman et al. [24] reported that an increase of serum creatinine was independently associated with 90-day mortality in AP. Lipinski et al. [25] noted that higher average values of Scr in patients on admission and 48 hrs later were related to a higher incidence of fatal AP. Muddana et al. [26] assumed that Scr levels may be less sensitive to small changes in the intravascular volume and better reflect visceral organ injury when comparing to BUN. In our study it emerged that Scr after 24 hours of hospitalization, compared to Scr at initial admission (0.76 versus 0.67), had a higher AUC (Figure 2). With a cut-off of 1.02 mg/dl, Scr at 24 hours of hospitalization achieved sensitivity of 51.7%, specificity of 90.2%, PPV of 34.1%, and NPV of 95.0%, meaning the sensitivity is low, though very specific (Table 2).

The mechanism of progression from a mild to a severe AP is induced by the release of proinflammatory cytokines (such as TNF-*α*, IL-6, and platelet activating factor) and the damage of reactive oxygen species (ROS) [27]. HDL-C can neutralize bacterial lipopolysaccharide and owns antioxidant and anti-inflammatory properties [7]. Unal et al. [28] reported that the activity of serum paraoxonase (the lipophilic antioxidant component of HDL-C) decreased in patients with AP. In this regard, it was hypothesized that impaired HDL-associated antioxidant defense may contribute to the severity of the disease [28]. Bugdaci et al. [29] reported that there was a significant inverse relation between HDL-C level, the length of hospitalization, and Ranson scoring in patients with AP. A small sample size (66 patients) study performed in ICU by Peng et al. [7] reported that low values of HDL were associated with high levels of inflammatory cytokines (TNF-*α*, IL-6), persistent organ failure, infected necrosis, and hospital mortality in subjects with severe AP. Our data indicated that HDL-C was a useful predictor of SAP (AUC 0.76) (Figure 2), with a cut-off of 22.4 mg/dl, achieving sensitivity of 51.7%, specificity of 90.5%, PPV of 34.9%, and NPV of 95.0% (Table 2).

As expected, the LR model that consists of the above three parameters markedly improved sensitivity. With a cut-off of −1.86, the LR model achieved an acceptable sensitivity of 62.7%, excellent specificity of 93.2%, PPV of 47.4%, and NPV of 96.1% (Table 2). As shown in the Fagan plot (Figure 3), if in a patient the LR model value was more than or equal to −1.86, the probability of developing SAP increased from 8.9% to 47%, and if the LR model value was less than −1.86, the probability decreased to 4%. Based on ROC analysis (Figure 2), the diagnostic performance of the LR mode (AUC 0.84) was superior to both BISAP score (AUC 0.82) and other predictors of SAP.

The strength points of this study include such a large sample size able to give the study a strong statistical power. Both patients in ICU and in general ward were enrolled in this study, thus reducing selection bias. According to our opinion, this is the first study in literature fully assessing Scr at 24 hours and validating HDL-C as a predictor of SAP, respectively, as well as determining the best cut-off value of HDL-C for prediction of severe acute pancreatitis. The developed LR model showed a good predictive performance in terms of discrimination and calibration. The LR model with a high AUC may be helpful to guide triage and to manage patient with AP. The limitation of our study was that BUN and Scr were measured at 24 hours after admission, which may influence the early application of our LR model in a subgroup of AP patients who rapidly developed progressive multiple organ failure in the first few days following the onset of acute pancreatitis (also recognized as fulminated acute pancreatitis in previous literature) [30]. On the other hand, even if it has been internally validated by bootstrap technique, testing the performance of our LR model in an external independent sample will be necessary in the future.

In conclusion, we have confirmed that HDL-C at admission and Scr at 24 hours may predict development of SAP. The LR model consisting of HDL-C at admission and BUN and Scr at 24 hours takes on a high diagnostic accuracy of prediction of development of SAP. It is an additional tool to stratify patients at risk of SAP and its application on admission may improve clinical care and strategies of management in acute pancreatitis.

## Figures and Tables

**Table 1:** Univariate analysis of predictive factors of acute pancreatitis in 647 patients.

Characteristic	Mild AP	Moderate AP	Severe AP	*p* value
	(*N* = 491)	(*N* = 98)	(*N* = 58)	
Median age, years (IQR)	47 (37–62)	47.5 (40–63)	51 (38–66)	0.165
Male sex, *N* (%)	309 (62.9)	66 (67.4)	31 (53.4)	0.219
BMI	23.4 (20.9–26.1)	24.6 (21.5–26.0)	24.2 (22.1–26.6)	0.048
Etiology				0.002
Biliary, *N* (%)	222 (45.2)	32 (32.7)	18 (31.0)	
Alcohol, *N* (%)	63 (12.8)	23 (23.5)	4 (6.9)	
Hypertriglyceridemia, *N* (%)	22 (4.5)	7 (7.1)	7 (12.1)	
Idiopathic, *N* (%)	184 (37.5)	36 (36.7)	29 (50.0)	
Laboratory findings				
Hematocrit	0.42 (0.38–0.45)	0.43 (0.39–0.47)	0.44 (0.40–0.47)	0.001
HDL-C (mg/dl)	41.3 (32.0–51.0)	36.7 (25.5–51.7)	22.4 (17.8–38.2)	<0.001
BUN, mg/dl (IQR)	13.2 (10.4–16.5)	12.2 (9.8–16.5)	19.9 (15.1–31.9)	<0.001
BUN (24 h), mg/dl (IQR)	12.9 (9.5–16.8)	12.2 (9.5–19.0)	26.0 (17.1–34.5)	<0.001
Creatinine, mg/dl (IQR)	0.72 (0.61–0.86)	0.72 (0.61–0.88)	0.92 (0.66–1.82)	<0.001
Creatinine (24 h), mg/dl (IQR)	0.72 (0.59–0.86)	0.68 (0.55–0.87)	1.04 (0.74–2.34)	<0.001
BISAP score	1 (0-1)	1 (1-2)	2 (1–3)	<0.001

IQR = Interquartile Range; *N* = number; AP = acute pancreatitis; BMI = Body Mass Index; HDL-C = High-Density Lipoprotein Cholesterol; BUN = Blood Urea Nitrogen; BISAP = Bedside Index for Severity in Acute pancreatitis.

**Table 2:** Diagnostic values of various predictors of severe acute pancreatitis.

Variable	Cut-off value	Sensitivity	Specificity	PPV	NPV
HDL-C	22.4 mg/dl	51.7	90.5	34.9	95
BUN	22.7 mg/dl	46.6	90.7	32.9	94.5
BUN (24 hrs)	21.8 mg/dl	56.9	90.2	36.3	95.5
Scr (24 hrs)	1.02 mg/dl	51.7	90.2	34.1	95
BISAP score	2	65.5	83.4	27.9	96.1
LR model	−1.86	62.7	93.2	47.4	96.1

HDL-C = High-Density Lipoprotein Cholesterol; BUN = Blood Urea Nitrogen; Scr = Serum creatinine; BISAP = Bedside Index for Severity in Acute pancreatitis; LR model = logistic regression model; PPV = positive predictive value; NPV = negative predictive value.
